# Derivation and Validation of The Prehospital Difficult Airway IdentificationTool (PreDAIT): A Predictive Model for Difficult Intubation

**DOI:** 10.5811/westjem.2017.1.32938

**Published:** 2017-04-17

**Authors:** Jestin N. Carlson, David Hostler, Francis X. Guyette, Mark Pinchalk, Christian Martin-Gill

**Affiliations:** * Allegheny Health Network, Department of Emergency Medicine, Erie, Pennsylvania; † University of Pittsburgh, Department of Emergency Medicine, Pittsburgh, Pennsylvania; ‡ University at Buffalo, Department of Exercise and Nutrition Sciences, Buffalo, New York; § University at Buffalo, Department of Emergency Medicine, Buffalo, New York; ¶ Pittsburgh Emergency Medical Services, Pittsburgh, Pennsylvania

## Abstract

**Introduction:**

Endotracheal intubation (ETI) in the prehospital setting poses unique challenges where multiple ETI attempts are associated with adverse patient outcomes. Early identification of difficult ETI cases will allow providers to tailor airway-management efforts to minimize complications associated with ETI. We sought to derive and validate a prehospital difficult airway identification tool based on predictors of difficult ETI in other settings.

**Methods:**

We prospectively collected patient and airway data on all airway attempts from 16 Advanced Life Support (ALS) ground emergency medical services (EMS) agencies from January 2011 to October 2014. Cases that required more than two ETI attempts and cases where an alternative airway strategy (e.g. supraglottic airway) was employed after one unsuccessful ETI attempt were categorized as “difficult.” We used a random allocation sequence to split the data into derivation and validation subsets. Using backward elimination, factors with a p<0.1 were included in the multivariable regression for the derivation cohort and then tested in the validation cohort. We used this model to determine the area under the curve (AUC), and the sensitivity and specificity for each cut point in both the derivation and validation cohorts.

**Results:**

We collected data on 1,102 cases with 568 in the derivation set (155 difficult cases; 27%) and 534 in the validation set (135 difficult cases; 25%). Of the collected variables, five factors were predictive of difficult ETI in the derivation model (adjusted odds ratio, 95% confidence interval [CI]): Glasgow coma score [GCS] >3 (2.15, 1.19–3.88), limited neck movement (2.24, 1.28–3.93), trismus/jaw clenched (2.24, 1.09–4.6), inability to palpate the landmarks of the neck (5.92, 2.77–12.66), and fluid in the airway such as blood or emesis (2.25, 1.51–3.36). This was the most parsimonious model and exhibited good fit (Hosmer-Lemeshow test p = 0.167) with an AUC of 0.68 (95% CI [0.64–0.73]). When applied to the validation set, the model had an AUC of 0.63 (0.58–0.68) with high specificity for identifying difficult ETI if ≥2 factors were present (87.7% (95% CI [84.1–90.8])).

**Conclusion:**

We have developed a simple tool using five factors that may aid prehospital providers in the identification of difficult ETI.

## INTRODUCTION

Airway management is a critical intervention in the prehospital resuscitation of specific patient populations. Endotracheal intubation (ETI) is a standard method of airway management, although its practice in the prehospital setting can be challenging.^1^ Multiple factors related to the austere environment and unscreened patient population make prehospital ETI more challenging than in other settings. As a result, a greater number of intubation attempts may be required, which have been associated with adverse events including hypoxia, bradycardia and even death.^2^,^3^

Supraglottic airways (SGA) and bag-valve-mask (BVM) ventilation can be valuable alternatives when ETI efforts are unsuccessful and may be used as first-line interventions in select populations if difficult ETI is anticipated.^4^,^5^ Proper identification of cases of potentially difficult ETI could allow providers to focus on alternative airway management strategies, thereby minimizing the risks associated with multiple or prolonged ETI attempts.^6^–^8^

Previous works have identified multiple factors associated with difficult ETI in a variety of acute care settings including the prehospital setting, intensive care unit, and emergency department.^9^–^13^ Although predictors and resultant treatment pathways have been identified, we are unaware of any externally validated, simplified identification tools for prehospital providers, identifying those factors most predictive of difficult ETI.^9^,^10^,^14^ Given the adverse events associated with ETI efforts, rapid identification of difficult ETI through such a tool could help to improve the safety of prehospital airway management. We sought to derive and validate a simplified tool to allow EMS providers to rapidly identify cases of difficult ETI.

## METHODS

### Study Design and Setting

We performed a prospective, observational study involving 16 ground emergency medical service (EMS) agencies to develop a predictive model for difficult ETI. These suburban and rural EMS agencies respond to approximately 100,000 EMS calls annually within a 10-county regional EMS system in Southwestern Pennsylvania. Advanced Life Support (ALS) ambulances for all participating EMS agencies are typically staffed with one paramedic who can perform advanced airway management including intubation and SGA placement and one emergency medical technician (EMT) who can perform basic airway management only. EMS providers function within statewide EMS protocols, which do not allow the performance of rapid sequence or sedation-assisted intubation. For patients in cardiac arrest, intubation may occur after an initial resuscitation period of 10 minutes during which basic airway management is emphasized, consistent with national guidelines. Providers receive an annual hands-on airway skills assessment, ongoing didactic education on airway management (approximately 1–2 hours/year), and typically perform 1–2 intubations/year.^15^ All participating EMS agencies receive medical oversight through the same healthcare system, coordinated through a single academic institution.

**Population Health Research Capsule**

What do we already know about this issue?

Previous factors have been associated with difficult intubation in the prehospital setting.

What was the research question?

We sought to prospectively derive and validate a tool to identify difficult intubation in the prehospital setting.

What was the major finding of the study?

A simple tool, using five factors (GCS>3, limited neck movement, trismus/jaw clenched, inability to palpate the landmarks of the neck, and fluid in the oropharynx), may aid prehospital providers in identifying difficult intubation.

How does this improve population health?

This tool may help to guide prehospital airway interventions.

### Selection of Participants

All patient-care documentation at these agencies is performed using a single National EMS Information System (NEMSIS)-compliant electronic patient care record (emsCharts, Warrendale, PA). Data were collected on all patients undergoing advanced airway management (intubation or supraglottic airway) by EMS during the study period. We excluded cases with an unknown number of ETI attempts, those where nasotracheal intubation was performed and those where a SGA was placed as the first advanced airway. There were no age, medical category, or other exclusionary criteria. This study had institutional review board approval.

### Methods and Measurements

All data were collected using a custom form in the electronic patient care report within emsCharts. The form was automatically activated for any case where an advanced airway procedure was documented. Medical providers were required to complete this form before the medical record could be finalized. Based on previous work evaluating difficult airways, the data elements in the form included patient demographics, patient characteristics, difficult airway characteristics, procedural characteristics, and techniques used to successfully intubate the patient (Table 1).^9^,^10^,^12^,^16^ Upon completion of the custom form, the form was automatically forwarded, without patient or agency identifiers, to the study investigators. We then entered data into a spreadsheet for data analysis (Microsoft Excel, Redmond, WA).

### Measurement Definitions

We defined difficult intubation (“difficult ETI”) as either more than two attempts at laryngoscopy or one unsuccessful attempt at laryngoscopy, followed by either SGA placement or BVM ventilation. Providers were not informed of this definition and were simply asked to report the number of attempts and device placed. As the exact age of patients may be unknown to providers in the prehospital setting, providers were asked to estimate the patient’s age by decile. Weight has previously been identified as a predictor of difficult ETI and although the exact weight is often unknown in the prehospital setting, EMS providers are often able to reliably estimate weight within 20% of actual weight.^17^ As a result, providers were asked to estimate the patient’s weight by categories. We collected difficult airway characteristics based on previous work, which included Glasgow coma score (GCS) >3; limited movement of the neck (e.g. cervical collar in place or “other”); gag reflex present; trismus/jaw clenched; neck or facial trauma; inability to palpate the landmarks of the neck (e.g., cricoid cartilage or thyromental distance); or fluid in the airway (e.g. blood or emesis).^9^,^10^,^12^,^16^,^18^ Providers were asked (yes or no) if they felt the intubation was difficult. Providers were also asked to generally classify the location of the ETI attempts (in the ambulance, at the scene on a stretcher, at the scene but not on a stretcher) and broadly categorize the status of the patient and indication for intubation at the time of ETI (cardiac arrest, medical condition not in cardiac arrest, traumatic arrest, traumatic condition not in arrest). Providers were asked to report the number of attempts at ETI and were informed that an attempt was defined as the passage of the laryngoscope past the lips.

### Analysis

Assuming 10% missing or inappropriately completed data entry and a difficult ETI rate of 20% (based on previous work with ETI data from these agencies), we determined we would need 1,200 cases total to evaluate a maximum of 10 variables in our multivariable model. This allowed for identification of a sufficient number of difficult ETI cases to develop a robust clinical decision tool without an exhaustive number of factors for providers to recall when using the prediction tool in the clinical setting.^19^ We split the data into derivation and validation subsets according to a random allocation sequence using the =RANDBETWEEN(0,1) function in Excel v 15.5.5 (Microsoft Corp, Redmond, WA).

We compared data between those defined as “difficult ETI” and “not difficult ETI.” A priori, we established a list of variables that have been shown to be associated with difficult ETI in a variety of settings. Recalling all elements from the list may be challenging for providers; therefore, we sought to include those with the greatest propensity for predicting difficult ETI, and using backward elimination we incorporated factors with a p<0.1 in the multivariable logistic regression for the derivation cohort. We also performed a sensitivity analysis examining alternative models that included all variables, those with both individual variables, and with combining similar variables (e.g., generating a new variable for limited neck movement including both patients with cervical collars and those with other causes of limited neck mobility). As pediatric patients represent a unique patient population where providers may not routinely perform ETI, we also retested the models, excluding pediatric patients.^20^,^21^ Receiver operating characteristics (ROC) curves were created to determine the area under the curve (AUC) for each model along with the sensitivity and specificity by number of factors present. We used the Hosmer-Lemeshow test to determine the goodness-of-fit for each model. We sought a model that would maximize specificity for the prediction of difficult ETI to allow providers to best identify those cases and tailor their approach to these difficult airways. The most parsimonious model maximizing specificity and having the greatest AUC was then applied to the validation set. We then calculated AUC, sensitivity and specificity for the validation set. All analyses were completed with Stata v 12 (Stata Corp, College Station, TX).

## RESULTS

### Characteristics of Study Subjects

**Figure 1:**
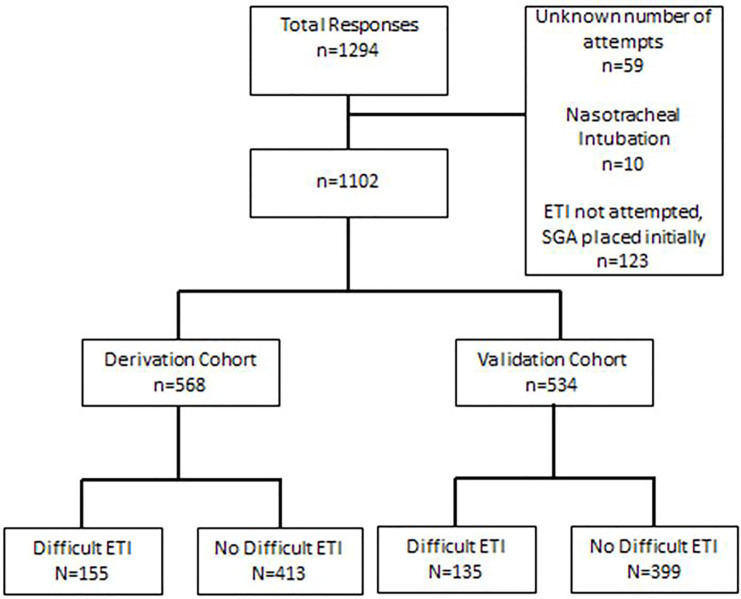
Study flow diagram of patients in study of factors indicative of difficult-airway identification. *ETI*, endotracheal intubation; *SGA*, supraglottic airway.

From January 2011 to October 2014, we collected data on 1,294 cases of which 1,102 were used for the derivation (n=558) and the validation (n=534) sets (Figure 1). Difficult ETI was identified in a similar proportion of the derivation (N=155, 27.3%) and validation (N=135, 25.3%) cohorts.

The proportion of patients estimated to have a weight >150kg was greater in the difficult ETI population of both the derivation and validation cohorts (Table 1). Age, patient status (e.g., cardiac arrest), and location of ETI were similar between those with and without difficult ETI in both cohorts. Patients with difficult ETI had a greater number of difficult airway characteristics (DAC) in both the derivation and validation cohorts [median (interquartile range)]: 2 (1–3) vs. 1 (0–2), and 2 (1–3) vs. 1 (0–2).] The majority of airways were successfully managed with ETI on the first attempt; however, approximately one in five airways were ultimately managed with a SGA in both the derivation and validation sets (Table 2).

### Main Results

**Figure 2:**
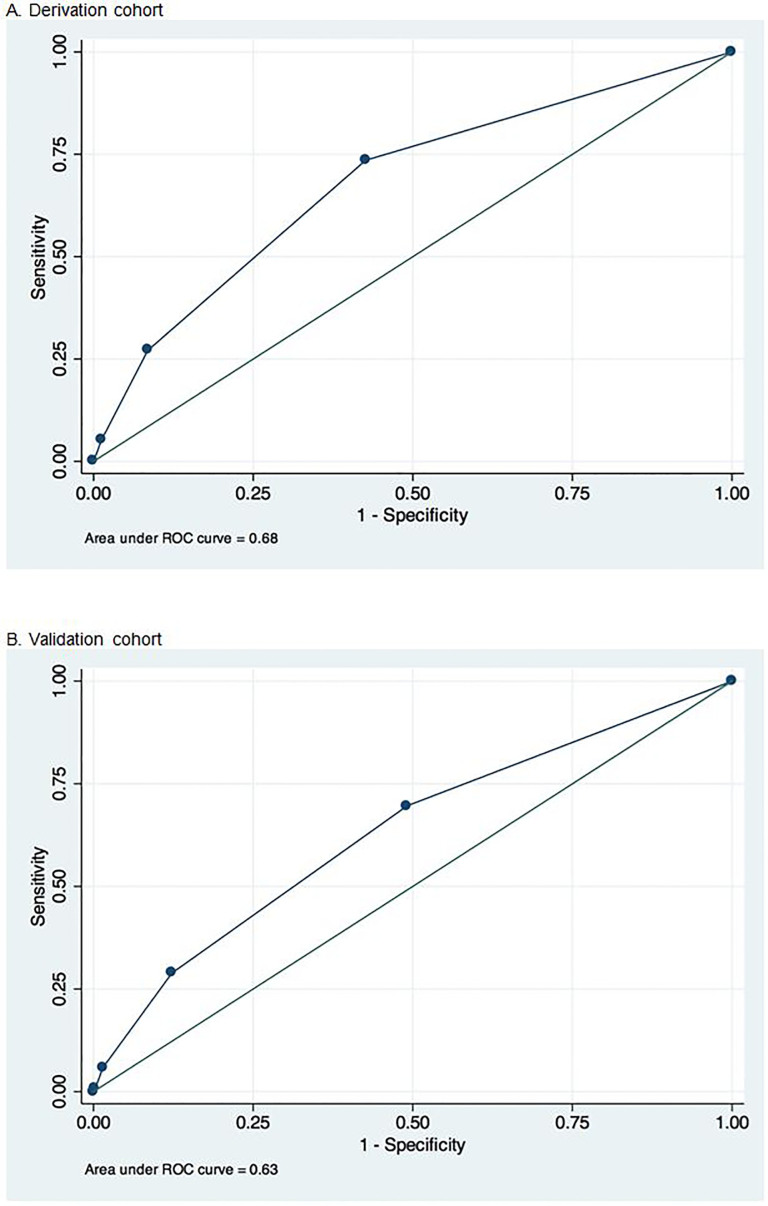
Receiver operator characteristic (ROC) curves for the derivation and validation cohorts in study to identify predictive factors for difficult airway.

Before combining any categories of variables, multiple variables were predictive of difficult ETI in various iterations of the model including GCS >3, trismus/jaw clenched, inability to palpate the landmarks of the neck, blood in the airway and emesis in the airway. After combining variables assessing neck mobility and those identifying fluids in the oropharynx (blood and emesis), five factors were predictive of difficult ETI in the derivation model (adjusted odds ratio, 95% CI): GCS>3 (2.15, 1.19–3.88), limited neck movement (2.24, 1.28–3.93) trismus/jaw clenched (2.24, 1.09–4.6), inability to palpate the landmarks of the neck (5.92, 2.77–12.66), and fluid in the oropharynx (2.25, 1.51–3.36) (Table 3). This was the most parsimonious of the tested models and exhibited good fit (Hosmer-Lemeshow test p = 0.167) with an AUC of 0.68 (95% CI [0.64–0.73]) (Figure 2). This model had 91.5% specificity (95% CI [88.4–94]) for identifying difficult ETI if ≥2 factors were present and 98.8% specificity (97.2–99.6) if ≥3 factors were present. As a result, this model was used for the validation cohort.

**Figure 3:**
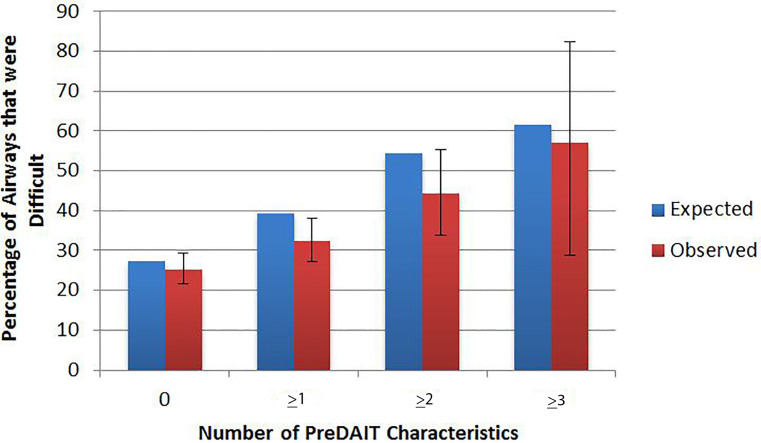
Calibration curve of the Prehospital Difficult Airway Identification Tool (PreDAIT).

When applied to the validation set, the model had an AUC of 0.63 (0.58–0.68) (Figure 2) and specificities of 87.7% (84.1–90.8) and 98.5% (96.8–99.4) for identifying difficult ETI when ≥2 or ≥3 factors were present respectively (Table 4). Over 70% of cases were correctly classified if ≥2 factors were present and calibrations curves were similar between observed and expected values (Figure 3). Removing pediatric patients did not significantly alter the sensitivity, specificity, or accuracy.

## LIMITATIONS

Our work had several limitations. While our data were collected prospectively, providers were required to complete a specific form for data collection and therefore were aware of the nature of the study. This may have introduced a reporting bias such as in the number of ETI attempts. Providers may have also been more likely to report difficult airway characteristics if the case required several attempts at intubation. However, it is possible this awareness helped to improve providers’ recognition of the presence of specific airway-related factors and the reporting of these factors. While we chose to measure the preselected variables, there may be other aspects of the scene (e.g., limited space around the patient, poor lighting, etc.) that may have influenced the provider’s decision to perform ETI. We did not assess these environmental factors. We did not evaluate provider-specific factors, such as provider experience or procedural competency. To ensure confidentiality during data collection, we did not identify the agency performing the ETI. As such, we were unable to assess for clustering in our analyses. We also were unable to determine if the same patient occurred multiple times within the dataset although we believe this would occur infrequently.

All intubations were performed by EMS personnel in Pennsylvania who perform a median of 1–2 intubations per year.^15^ Our provider population consists of ALS, ground-based agencies that are not permitted to use medications to facilitate ETI (e.g. rapid sequence intubation [RSI]). RSI may be available to select providers in specific areas but is not universally available to ALS providers. We believe our setting, where RSI is not available, to be similar to many EMS settings in the United States, although future work will be needed to examine this scoring system in agencies with RSI capabilities. We did not include cases where ETI was not attempted, and therefore did not collect data on these cases. In cases where only SGAs or BVM were used it is unknown if the airway was managed with these techniques because the provider anticipated the airway to be difficult for some other reason. As we did not collect data on these cases, we were unable to include them in our model.

There may be select populations where further refinement of this tool is required. For example, a low percentage of our intubation attempts were in pediatric patients with distinct anatomy. Pediatric ETI is a relatively infrequent event, occurring in <1% of pediatric EMS responses, and previous work has questioned the utility of pediatric intubation in the prehospital setting.^20^,^21^ Further work will be needed to evaluate this tool in specific populations such as pediatric patients. Also, a large proportion of patients in our study sample were in cardiac arrest, which limited assessment of patients not in cardiac arrest. However, we believe this cohort accurately represents the majority of out-of-hospital patients intubated by EMS providers without RSI capabilities. ETI is a technically challenging skill with a steep learning curve that requires continued practice to maintain proficiency.^22^ Future work may be needed to assess this prehospital difficult airway identification tool, in specific provider populations.

## DISCUSSION

ETI in the prehospital setting is complicated by several factors including the austere environment, provider experience, and the critically ill patient population. ETI is one of the most common procedures in critically ill prehospital patients, occurring in 8–10/1,000 EMS responses with an overall success rate of 77%.^20^,^23^,^24^ Using this internally validated tool, prehospital providers can predict difficult ETI cases with over 87% specificity if ≥2 of the following characteristics are present: GCS>3; limited neck movement; trismus/jaw clenched; inability to palpate the landmarks of the neck; and fluid in the oropharynx. We are unaware of any previous efforts to design such a tool for predicting difficult ETI in the prehospital setting. While there have been several publications describing predictors of difficult intubation, none have been derived and validated as a predictive model for use by prehospital providers.^13^,^25^ A tool developed by a group of French anesthesiologists was evaluated in a small prehospital subgroup; however, generalization is limited as intubations were all performed by emergency physicians.^26^

Previous work has helped to identify several characteristics that, in isolation, may help to predict difficult airways. These include blood in the airway, vomit in the airway, short neck, c-spine immobility, short mandible, obesity, airway edema, facial trauma, large tongue, limited Mallampati score, intra-incisor distance of <3 fingers, and thyromental distance of <2 fingers.^9^,^10^,^13^,^27^ These studies, examining multiple characteristics, provided the basis for the factors selected in our analysis.

A previously published retrospective analysis examined 61 factors associated with unsuccessful ETI in the prehospital setting and identified several predictive factors including trismus/jaw clenched, weight, and the presence of a gag reflex.^10^ While trismus/jaw clenched contributed to our model, weight and the presence of a gag reflex did not. Weight and gag have been identified in other studies and may still represent important characteristics when assessing patients requiring airway management in the prehospital setting.^9^ Weight, for example, was significant in the univariate analysis, however did not appreciably contribute to the model. As such, weight and other factors were not included in the final model for our simplified difficult airway identification tool. Other identified factors included “inability to pass the endotracheal tube through the cords” and “inability to visualize the cords,” although these incorporate aspects of laryngoscopy and occur after the provider has made the decision to perform ETI.^10^ As a result, we did not include these factors in our model.

Incorporating difficult airway characteristics into a simplified, rapid, validated tool may help providers better identify this population *before* attempting ETI, thereby minimizing the risks associated with ETI. A rapid evaluation of patients for the aspects of this Prehospital Difficult Airway Identification Tool, or PreDAIT, may help providers better assess the potential for difficult intubation and manage the airway by other means. While this tool may be helpful in identifying those patients most likely to be a difficult intubation, (>87% specificity if ≥2 factors are present), difficult ETI cases can and do occur in patients with no PreDAIT characteristics. Of the difficult airway cases in the validation cohort, 30% had none of the five factors, highlighting the challenges with identifying *all* cases of difficult ETI. Although this tool identifies patients at greatest risk for unsuccessful ETI in our population, providers must still anticipate challenges with ETI and be facile with alternative management techniques in the event of unsuccessful ETI efforts.

We feel this tool may be helpful with the timing of airway interventions and could be used by medical directors to refine prehospital airway management guidelines. For example, in cases where zero PreDAIT factors are present, directors may suggest performing ETI per standard protocols. In cases with one identified PreDAIT factor, providers may still perform ETI but have adjuncts readily available in the event of unsuccessful initial ETI attempts. In situations with two or more identified PreDAIT factors, alternate airway management could be recommended (e.g. using a SGA as the initial airway management strategy, awaiting critical care providers with advanced techniques such as video laryngoscopy and/or RSI, or performing BVM ventilation until hospital arrival).

While ETI by direct laryngoscopy has long been used as the primary method of airway management, several other alternatives exist such as BVM ventilation, non-invasive positive pressure ventilation, video laryngoscopy (VL) and SGAs. VL has been advocated as a means of improving intubation success and may be a valuable adjunct in difficult airway cases.^28^–^31^ While our work was not designed to determine the impact of VL on intubation outcomes due to low use of VL in our area (only 3.5% of cases), the positive impact of VL on ETI success in other studies highlights the potential utility of VL in cases where patients have several identified PreDAIT factors (i.e., greater probability of difficult ETI). Similarly, SGAs have successfully been used in the prehospital setting as first-line interventions in select populations^4^,^5^ and in cases of unanticipated difficult airways.^14^ While our work identifies variables predictive of difficult ETI, previous work has found that similar variables such as presence of a gag reflex are also associated with unsuccessful SGA placement.^16^ In combination with our tool, providers may consider this and elect to defer advanced airway maneuvers (ETI or SGA) in the prehospital setting if in proximity to the hospital.

## CONCLUSION

We prospectively derived and internally validated a simple tool identifying five factors predictive of difficult ETI: GCS>3; limited neck movement; trismus/jaw clenched; inability to palpate the landmarks of the neck; and fluid in the oropharynx. The PreDAIT may help providers identify difficult ETI in the prehospital setting. Future studies should externally validate this model in other EMS systems.

## Figures and Tables

**Table 1:** Patient and airway demographics.

Characteristic	Total derivation n=568 (%)	Difficult ETI n=155	Not difficult ETI n=413	Total validation n=534	Difficult ETI n=135	Not difficult ETI n=399
Age (years)	n=560			n=529		
<10	12 (2)	5 (3)	7 (2)	5 (1)	0 (0)	5 (1)
10–19	8 (1)	1 (1)	7 (2)	13 (2)	4 (3)	9 (2)
20–29	9 (2)	0 (0)	9 (2)	16 (3)	2 (1)	14 (4)
30–39	31 (6)	10 (6)	21 (5)	21 (4)	8 (6)	13 (3)
40–49	41 (7)	17 (11)	24 (6)	46 (9)	15 (11)	31 (8)
50–59	93 (17)	29 (19)	64 (16)	94 (18)	37 (27)	57 (14)
60–69	120 (21)	37 (24)	83 (20)	112 (21)	30 (22)	82 (21)
70–79	111 (20)	26 (17)	85 (21)	108 (20)	21 (16)	87 (22)
>79	135 (24)	29 (19)	106 (26	114 (22)	18 (13)	96 (24)
Gender, male	331 (58)	93 (60)	238 (58)	318 (60)	95 (70)	223 (56)
Weight (kilograms)	n=561			n=530		
<100	265 (47)	60 (39)	205 (50)	265 (48)	50 (38)	206 (52)
100–150	235 (42)	68 (45)	167 (41)	224 (42)	66 (50)	158 (40)
>150	61 (11)	24 (16)	37 (9)	50 (9)	17 (13)	33 (8)
Patient status	n=551			n=521		
Cardiac arrest	440 (80)	121 (82)	319 (79)	416 (80)	110 (83)	306 (79)
Medical condition (not in cardiac arrest)	73 (13)	15 (10)	58 (14)	67 (13)	13 (10)	54 (14)
Traumatic arrest	22 (4)	6 (4)	16 (4)	20 (4)	5 (4)	15 (4)
Traumatic condition (not in arrest)	16 (3)	6 (4)	10 (2)	18 (3)	5 (4)	13 (3)
Location of ETI	n=516			n=498		
Ambulance	242 (47)	67 (50)	175 (46)	236 (47)	56 (46)	180 (48)
Scene, not on a stretcher	252 (49)	61 (46)	191 (50)	247 (50)	61 (50)	186 (49)
Scene, on a stretcher	22 (4)	6 (4)	16 (4)	15 (3)	5 (4)	10 (3)
DACs						
Median total DACs (IQR)	1 (0–2)	2 (1–3)	1 (0–2)	1 (0–2)	2 (1–3)	1 (0–2)
Provider perceived ETI as difficult	244 (43)	135 (87)	109 (26)	224 (42)	108 (80)	116 (29)
None	257 (45)	37 (24)	220 (53)	235 (44)	41 (31)	194 (49)
GCS>3	57 (10)	23 (15)	34 (8)	49 (9)	10 (7)	39 (10)
Limited neck movement						
Cervical collar in place	19 (3)	8 (5)	11 (3)	30 (6)	8 (6)	22 (6)
Other limited neck mobility (e.g. kyphosis)	52 (9)	25 (16)	27 (7)	57 (11)	28 (21)	29 (7)
Gag reflex present	46 (8)	12 (8)	34 (8)	18 (3)	1 (1)	17 (4)
Trismus, jaw clenched	37 (7)	16 (10)	21 (5)	41 (8)	17 (13)	24 (6)
Neck or facial trauma	9 (2)	3 (2)	6 (1)	4 (1)	2 (1)	2 (1)
Unable to palpate landmarks of the neck	34 (6)	22 (14)	12 (3)	27 (5)	10 (7)	17 (4)
Fluid in the airway						
Blood	63 (11)	27 (17)	36 (9)	67 (13)	22 (16)	45 (11)
Emesis	143 (25)	54 (35)	89 (22)	167 (31)	66 (49)	101 (25)
Number of PreDAIT factors						
0	278 (49)	41 (27)	237 (57)	244 (46)	41 (30)	203 (51)
1	213 (38)	72 (46)	141 (34)	202 (38)	55 (41)	147 (37)
2	64 (11)	34 (22)	30 (7)	74 (14)	31 (23)	43 (11)
3	13 (2)	8 (5)	5 (1)	12 (2)	7 (5)	5 (1)
4	0	0	0	2 (<1)	1 (1)	1 (<1)
5	0	0	0	0	0	0

*ETI,* endotracheal intubation; *DAC*, difficult airway characteristics; *IQR*, interquartile range; *GCS*, glasgow coma scale; *PreDAIT*, Prehospital Difficult Airway Identification Tool.

Percent totals may not equal 100% due to rounding.

**Table 2:** Number of attempts and successful airway for the derivation and validation cohorts.

	Derivation, n = 568	Validation, n =534
Number of attempts		
1	343 (60)	323 (61)
2	157 (28)	156 (29)
>2	68 (12)	55 (10)
Median number of attempts (IQR)	1 (1 – 1)	1 (1 – 2)
Successful airway		
None	64 (11)	49 (9)
ETI	386 (69)	372 (70)
SGA	110 (20)	109 (21)

*ETI*, endotracheal intubation; *SGA*, supraglottic airway.

**Table 3:** Odds ratios for variables identified in the derivation cohort.

Characteristic	OR (95% CI)	p-value
Unadjusted univariate odds ratios for all predictor variables in the derivation cohort		
Age (decile)	0.92 (0.83–1.01)	0.083
Gender		
Male	Referent	
Female	1.1 (0.76–1.6)	0.61
Weight	1.46 (1.11–1.92)	0.007
Patient status		
Cardiac arrest	Referent	
Medical condition (not in cardiac arrest)	0.68 (0.37–1.25)	0.215
Traumatic arrest	0.99 (0.38–2.59)	0.918
Traumatic condition (not in arrest)	1.58 (0.56–4.45)	0.385
Location of ETI		
Ambulance	Referent	
Scene, not on a stretcher	0.83 (0.56–1.25)	0.378
Scene, on a stretcher	0.98 (0.37–2.61)	0.967
DACs		
None	0.28 (0.18–0.42)	<0.001
GCS>3	1.94 (1.1–3.42)	0.021
Limited neck movement	2.79 (1.64–4.72)	<0.001
Cervical collar in place	1.99 (0.78–5.04)	0.147
Other limited neck mobility (e.g. kyphosis)	2.75 (1.54–4.91)	0.001
Gag reflex present	0.94 (0.47–1.86)	0.849
Trismus, jaw clenched	2.15 (1.09–4.24)	0.027
Neck or facial trauma	1.34 (0.33–5.42)	0.683
Unable to palpate landmarks of the neck	5.53 (2.66–11.47)	<0.001
Fluid in the airway	2.25 (1.53–3.29)	<0.001
Blood	2.21 (1.29–3.78)	0.004
Emesis	1.95 (1.3–2.92)	0.001
Adjusted odds ratios for variables identified in the derivation cohort		
GCS>3	2.15 (1.19–3.88)	0.011
Limited neck movement	2.24 (1.28–3.93)	0.005
Trismus, jaw clenched	2.24 (1.09–4.6)	0.028
Unable to palpate landmarks of the neck	5.92 (2.77–12.66)	<0.001
Fluid in the airway (e.g. blood, emesis or both)	2.25 (1.51–3.36)	<0.001

*OR*, odds ratio, *CI*, confidence interval; *ETI*, endotracheal intubation; *DAC*, difficult airway characteristics; *GCS*, Glasgow coma scale.

**Table 4:** Details of the derivation and validation cohorts.

Cutpoint	Sensitivity (95% CI)	Specificity (95% CI)	Correctly classified	AUC (95% CI)
Derivation cohort*				
≥0	100 (97.6–100)	0 (0–0.9)	27.29	0.68 (0.64–0.73)
≥1	73.5 (65.9–80.3)	57.4 (52.5–62.2)	61.8	
≥2	27.1 (20.3–34.8)	91.5 (88.4–94)	73.94	
≥3	5.2 (2.3–9.9)	98.8 (97.2–99.6)	73.24	
Validation cohort#				
≥0	100 (97.3–100)	0 (0–0.9)	25.28	0.63 (0.58–0.68)
≥1	69.6 (61.1–77.2)	50.9 (45.9–55.9)	55.62	
≥2	28.9 (21.4–37.3)	87.7 (84.1–90.8)	72.85	
≥3	5.9 (2.6–11.3)	98.5 (96.8–99.4)	75.09	
≥4	0.7 (0–4.1)	99.7 (98.6–100)	74.72	

*AUC*, area under the curve; *CI*, confidence interval.

* No patients in the derivation cohort had 4 or 5 factors.

# No patients in the validation cohort had 5 factors.

## References

[1] Pepe PE, Copass MK, Joyce TH (1985). Prehospital endotracheal intubation: rationale for training emergency medical personnel. Ann Emerg Med.

[2] Wang HE, Lave JR, Sirio CA (2006). Paramedic intubation errors: isolated events or symptoms of larger problems?. Health Aff.

[3] Mort TC (2004). Emergency tracheal intubation: complications associated with repeated laryngoscopic attempts. Anesth Analg.

[4] Braude D, Richards M (2007). Rapid sequence airway (RSA)--a novel approach to prehospital airway management. Prehosp Emerg Care.

[5] Braude D, Southard A, Bajema T (2010). Rapid sequence airway using the LMA-Supreme as a primary airway for 9 h in a multi-system trauma patient. Resuscitation.

[6] Frascone RJ, Wewerka SS, Burnett AM (2013). Supraglottic airway device use as a primary airway during rapid sequence intubation. Air Med J.

[7] Chira S, Miller LG (2010). Staphylococcus aureus is the most common identified cause of cellulitis: a systematic review. Epidemiol and Infect.

[8] Fouche PF, Simpson PM, Bendall J (2014). Airways in out-of-hospital cardiac arrest: systematic review and meta-analysis. Prehosp Emerg Care.

[9] Gaither JB, Stolz U, Ennis J (2015). Association between difficult airway predictors and failed prehosptial endotracheal intubation. Air Med J.

[10] Wang HE, Kupas DF, Paris PM (2003). Multivariate predictors of failed prehospital endotracheal intubation. Acad Emerg Med.

[11] De Jong A, Molinari N, Terzi N (2013). Early identification of patients at risk for difficult intubation in the intensive care unit: development and validation of the MACOCHA score in a multicenter cohort study. Am J Respir Crit Care Med.

[12] Reed MJ, Dunn MJ, McKeown DW (2005). Can an airway assessment score predict difficulty at intubation in the emergency department?. Emerg Med J.

[13] Gaither JB, Spaite DW, Stolz U (2014). Prevalence of difficult airway predictors in cases of failed prehospital endotracheal intubation. J Emerg Med.

[14] Combes X, Jabre P, Margenet A (2011). Unanticipated difficult airway management in the prehospital emergency setting: prospective validation of an algorithm. Anesthesiology.

[15] Wang HE, Abo BN, Lave JR (2007). How would minimum experience standards affect the distribution of out-of-hospital endotracheal intubations?. Ann Emerg Med.

[16] Martin-Gill C, Prunty HA, Ritter SC (2015). Risk factors for unsuccessful prehospital laryngeal tube placement. Resuscitation.

[17] Axelband J, Malka A, Jacoby J (2004). Can emergency personnel accurately estimate adult patient weights?. Ann Emerg Med.

[18] Acker CG, Johnson JP, Palevsky PM (1998). Hyperkalemia in hospitalized patients: causes, adequacy of treatment, and results of an attempt to improve physician compliance with published therapy guidelines. Arch Intern Med.

[19] Cook CE (2008). Potential pitfalls of clinical prediction rules. J Man Manip Ther.

[20] Carlson JN, Gannon E, Mann NC (2015). Pediatric out-of-hospital critical procedures in the United States. Pediatr Crit Care Med.

[21] Gausche M, Lewis RJ, Stratton SJ (2000). Effect of out-of-hospital pediatric endotracheal intubation on survival and neurological outcome: a controlled clinical trial. JAMA.

[22] Wang HE, Seitz SR, Hostler D (2005). Defining the learning curve for paramedic student endotracheal intubation. Prehosp Emerg Care.

[23] Wang HE, Mann NC, Mears G (2011). Out-of-hospital airway management in the United States. Resuscitation.

[24] Carlson JN, Karns C, Mann NC (2016). Procedures performed by emergency medical services in the United States. Prehosp Emerg Care.

[25] Breckwoldt J, Klemstein S, Brunne B (2011). Difficult prehospital endotracheal intubation - predisposing factors in a physician based EMS. Resuscitation.

[26] Adnet F, Borron SW, Racine SX (1997). The intubation difficulty scale (IDS): proposal and evaluation of a new score characterizing the complexity of endotracheal intubation. Anesthesiology.

[27] Brown CA, Bair AE, Pallin DJ (2015). Techniques, success, and adverse events of emergency department adult intubations. Ann Emerg Med.

[28] Sakles JC, Patanwala AE, Mosier JM (2014). Comparison of video laryngoscopy to direct laryngoscopy for intubation of patients with difficult airway characteristics in the emergency department. Intern Emerg Med.

[29] Sakles JC, Mosier J, Chiu S (2012). A comparison of the C-MAC video laryngoscope to the Macintosh direct laryngoscope for intubation in the emergency department. Ann Emerg Med.

[30] Guyette FX, Farrell K, Carlson JN (2013). Comparison of video laryngoscopy and direct laryngoscopy in a critical care transport service. Prehosp Emerg Care.

[31] Wayne MA, McDonnell M (2010). Comparison of traditional versus video laryngoscopy in out-of-hospital tracheal intubation. Prehosp Emerg Care.

